# *Streptomyces* development is involved in the efficient containment of viral infections

**DOI:** 10.1093/femsml/uqad002

**Published:** 2023-01-16

**Authors:** Tom Luthe, Larissa Kever, Sebastian Hänsch, Aël Hardy, Natalia Tschowri, Stefanie Weidtkamp-Peters, Julia Frunzke

**Affiliations:** Institute of Bio- and Geosciences, IBG-1: Biotechnology, Forschungszentrum Jülich, 52425 Jülich, Germany; Institute of Bio- and Geosciences, IBG-1: Biotechnology, Forschungszentrum Jülich, 52425 Jülich, Germany; Center for Advanced Imaging, Heinrich Heine Universität Düsseldorf, 40225 Düsseldorf, Germany; Institute of Bio- and Geosciences, IBG-1: Biotechnology, Forschungszentrum Jülich, 52425 Jülich, Germany; Institute of Microbiology, Leibniz Universität Hannover, 30419 Hannover, Germany; Center for Advanced Imaging, Heinrich Heine Universität Düsseldorf, 40225 Düsseldorf, Germany; Institute of Bio- and Geosciences, IBG-1: Biotechnology, Forschungszentrum Jülich, 52425 Jülich, Germany

**Keywords:** *Streptomyces*, development, bacteriophages, phage defense, sporulation, viral infection

## Abstract

The formation of plaques represents the hallmark of phage infection visualizing the clearance of the bacterial lawn in structured environments. In this study, we have addressed the impact of cellular development on phage infection in *Streptomyces* undergoing a complex developmental life cycle. Analysis of plaque dynamics revealed, after a period of plaque size enlargement, a significant regrowth of transiently phage-resistant *Streptomyces* mycelium into the lysis zone. Analysis of *Streptomyces venezuelae* mutant strains defective at different stages of cellular development indicated that this regrowth was dependent on the onset of the formation of aerial hyphae and spores at the infection interface. Mutants restricted to vegetative growth (Δ*bldN*) featured no significant constriction of plaque area. Fluorescence microscopy further confirmed the emergence of a distinct zone of cells/spores with reduced cell permeability towards propidium iodide staining at the plaque periphery. Mature mycelium was further shown to be significantly less susceptible to phage infection, which is less pronounced in strains defective in cellular development. Transcriptome analysis revealed the repression of cellular development at the early stages of phage infection probably facilitating efficient phage propagation. We further observed an induction of the chloramphenicol biosynthetic gene cluster highlighting phage infection as a trigger of cryptic metabolism in *Streptomyces*. Altogether, our study emphasizes cellular development and the emergence of transient phage resistance as an important layer of *Streptomyces* antiviral immunity.

## Introduction

Bacteria are abundant in soil environments where spatial movement is limited and microbial density and diversity are higher than in most other habitats (Fierer 2017, Thompson et al. 2017, Rodriguez-R et al. 2018). In need for nutrients and space, while in constant contact with competitors and predators, soil bacteria developed different strategies to promote survival and distribution. Bacteria of the genus *Streptomyces* are members of the large phylum of Actinobacteria thriving in soil habitats (van Bergeijk et al. 2020). *Streptomyces* are well-known for their production of a wide range of clinically and industrially relevant secondary metabolites (Demain 1999, Watve et al. 2001) as well as their complex multicellular development (Flärdh and Buttner 2009, Barka et al. 2016, Chater 2016).

The most abundant predator in almost every environment, including the soil, are bacteriophages (phages) (Williamson et al. 2017). Phages are viruses infecting bacteria and as such pose a constant threat to bacterial populations, playing a major role in microbial evolution and community dynamics (Clokie et al. 2011, Chevallereau et al. 2021). The research on phages infecting members of the Actinobacteria (actinobacteriophages) has seen enormous efforts in isolating, sequencing and characterizing on many associated host genera including 25 *Streptomyces* species. The over 4000 sequenced genomes curated at The Actinobacteriophage Database (phagesdb.org) reveal an immense diversity of actinobacteriophages regarding genome size, morphology, lifestyle, host range and genomic equipment, but display considerable bias towards the species of *Mycobacterium smegmatis* with more than 2000 sequenced phages (Hatfull 2020). Despite this wealth of data, still little is known about phage-bacteria interactions in a host capable of secondary metabolite production and multicellular development.

In contrast to classical unicellular bacteria, *Streptomyces* do not follow the typical binary fission style of reproduction but undergo a complex life cycle starting from single spores developing into a branched vegetative mycelium with connected compartments that is followed by the erection of unbranched aerial hyphae growing into the air under unfavorable conditions (Elliot and Talbot 2004, Flärdh 2010). From aerial hyphae, chains of unigenomic spores are generated and dispersed (Sigle et al. 2015). During progression of the life cycle, a programmed cell death-like mechanism provides resources for the following developmental steps (Miguélez, Hardisson and Manzanal 1999, Manteca, Fernandez and Sanchez 2006). More precisely, coordinated cell death among the old vegetative mycelium precedes the erection of aerial hyphae (Manteca et al. 2007, Tenconi et al. 2018). In order to protect carbon and nitrogen resources from being captured by other soil-living organisms, production of secondary metabolites such as antibiotics is coupled to developmental processes (Rigali et al. 2008). Recent studies revealed that—besides their antibacterial properties—some of these natural products may actually confer protection against a variety of different phages. This was exemplified by DNA-intercalating molecules of the class of anthracyclines as well as for different aminoglycosides produced by *Streptomyces* (Kronheim et al. 2018, Hardy, Kever and Frunzke 2022, Kever et al. 2022). While first studies suggest a diverse repertoire of antiviral molecules produced by these bacteria, the impact of the developmental peculiarities and multicellular lifestyle of *Streptomyces* on phage-host interactions has not been addressed so far.

*Streptomyces* multicellular development is governed by a complex regulatory network. Part of this network are *bld* and *whi* genes encoding regulators, which control genes involved in key developmental steps. The so-called ‘master regulator’ BldD controls more than 160 different targets in the *Streptomyces* genome and is itself regulated by c-di-GMP (McCormick and Flärdh 2012, Tschowri et al. 2014, Bush et al. 2015). In the model strain *Streptomyces venezuelae*, BldD inhibits the expression of the sigma factor BldN, which activates *rdlABC* and *chpBCEFGH* genes encoding rodlins and chaplins necessary for building the hydrophobic sheath (Bibb et al. 2012). Together with SapB, the amyloid layer of hydrophobic rodlins and chaplins is necessary for the escape of aerial hyphae from the aqueous environment. Another target repressed by BldD is *whiB*, which, together with WhiA, stops tip growth of aerial hyphae by blocking expression of *filP*. WhiB activates septation, segregation and cell division in the filaments during transformation from aerial mycelium to spore chains by upregulation of *ftsK, parAB* and *ftsZ* (Bush et al. 2013, 2016). While WhiB-like proteins are widespread in Actinobacteria, they were recently shown to be common among actinobacteriophages too. Indeed, 24% of analyzed genomes encode *whiB* homologs, making WhiB-like genes the most abundant transcriptional regulators in actinobacteriophages (Sharma et al. 2021). Besides WhiB-like proteins, actinobacteriophages harbor several other genes potentially involved in the manipulation of cellular development, including *parB, ftsK*, or *ssgA* (Hardy et al. 2020). Given the genetic equipment of actinobacteriophages and the complex life cycle of their host it is likely that phages have evolved various mechanisms to manipulate development and that cellular development is an integral part of the multicellular antiphage defense strategies employed by *Streptomyces*.

In this study, we investigated the impact of phage infection on *Streptomyces* development using *S. venezuelae* as a model host. We observed that phage infection on solid media triggered the development of aerial hyphae and sporulation at the infection interface of the plaque. Analysis of mutant strains defective at different stages of *Streptomyces* development further confirmed the importance of cellular development for the establishment of phage-resistance phenotypes promoting the containment of viral infections. Global transcriptome analysis further suggested a suppression of cellular development during the early stages of phage infection.

## Results

### Impact of phage infection on *Streptomyces* development

**Figure 1.**
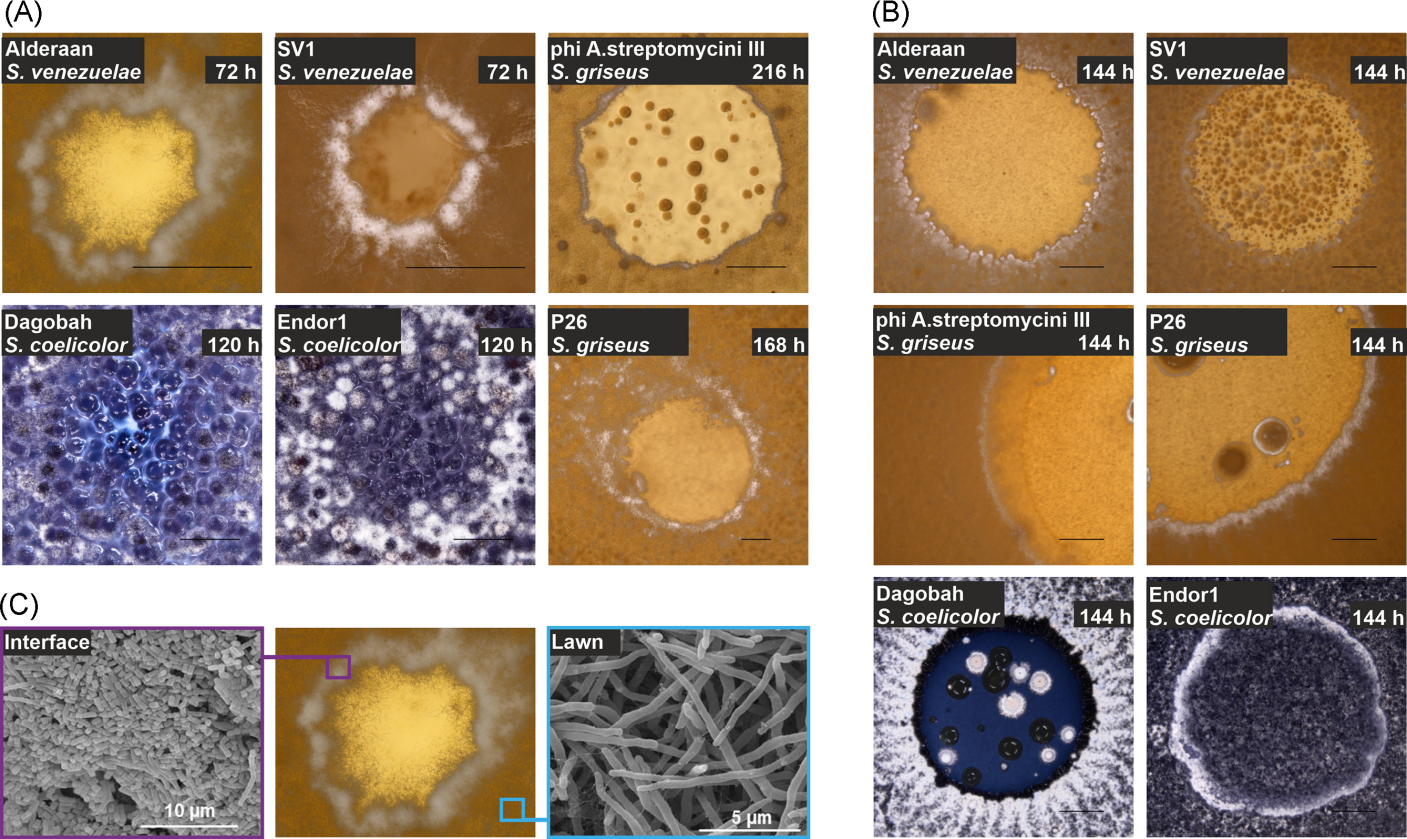
Phage infection leads to enhanced formation of aerial hyphae and spores at the plaque interface. (**A**) Stereo microscopy of representative plaques of the phages Alderaan and SV1 infecting *S. venezuelae*, phi A.streptomycini III and P26 infecting *S. griseus* and Dagobah and Endor1 infecting *S. coelicolor*. Scale bars represent 500 μm. (**B**) Stereo microscopy of spots of the six phage-host pairs. Scale bars represent 2 mm. (**C**) Scanning electron micrographs of samples from *S. venezuelae* infected by Alderaan taken from the plaque interface (purple) or lawn (cyan).

To investigate *Streptomyces* development during bacteriophage infection, we monitored plaque formation of six phages (Alderaan, SV1, phi A.streptomycini III, P26, Dagobah and Endor1, see Table S1) on the lawn of three different *Streptomyces* species (*S. venezuelae, S. griseus*, and *S. coelicolor*, see Table S2) using stereo microscopy (Fig. 1A). While significant differences were observed with regard to the spatiotemporal dynamics of plaque growth and cellular development, phage infection resulted for all six phage-host pairs in an enhanced formation of aerial hyphae at the infection interface. An even more pronounced phenotype was observed when spotting high titers of phages on plates leading to massive cell lysis and enhanced cellular development at the spot interface (Fig. 1B). Scanning electron microscopy of *S. venezuelae* infected with Alderaan confirmed the formation of spores at the plaque interface, whereas the unaffected outskirts of the bacterial lawn showed mainly vegetative mycelium and only few spore chains under the tested conditions (Fig. 1C).

### *S. venezuelae* development promotes the emergence of transient phage resistance

Since *S. venezuelae* is an established model system for studying cellular development, we focused on plaque growth and dynamics of phage Alderaan in the following.

**Figure 2.**
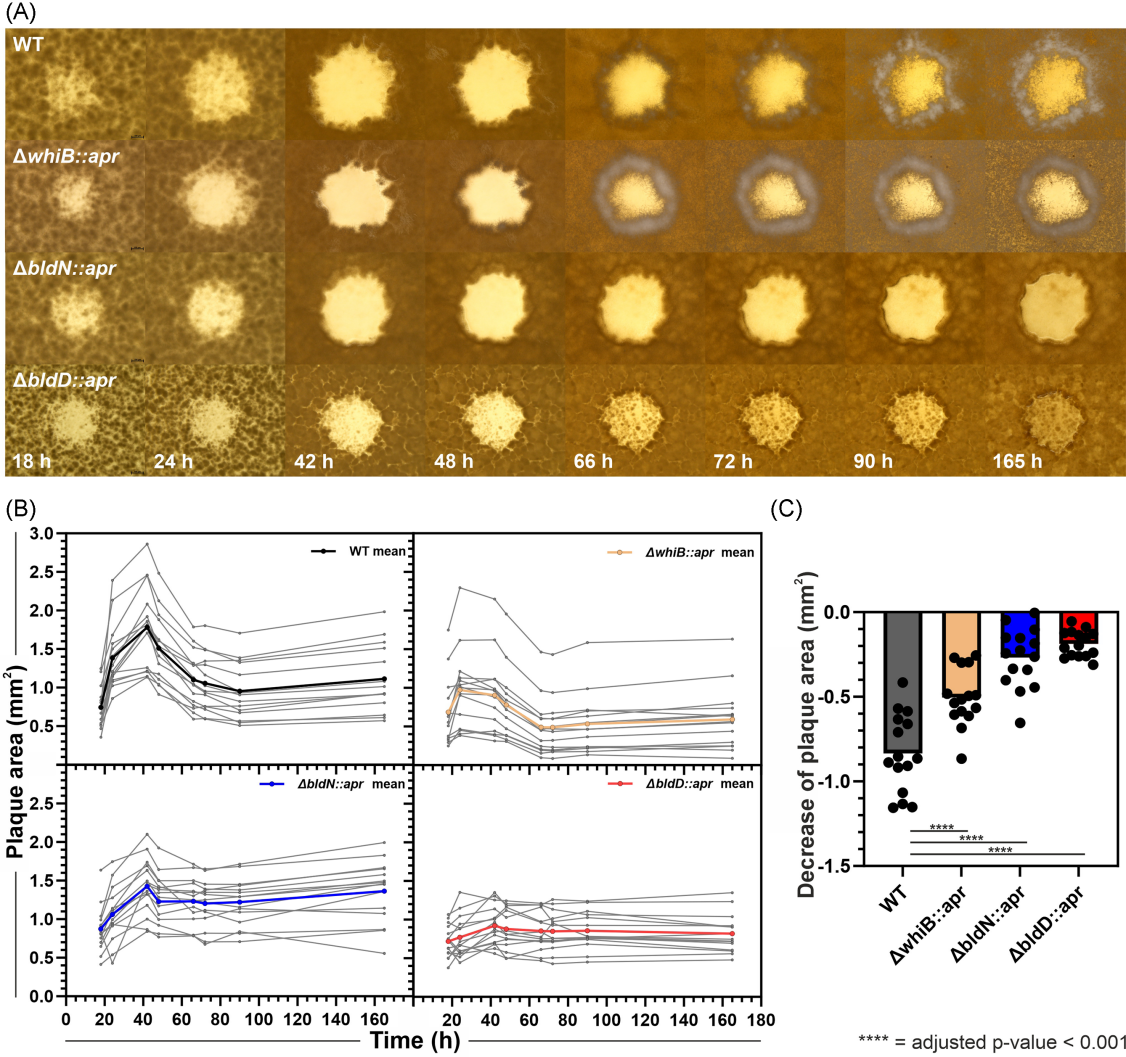
Plaque shrinkage in *S. venezuelae* relies on the formation of aerial hyphae and spores. Imaging and measuring of Alderaan plaques on *S. venezuelae* NRRL B-65442 WT and developmentally impaired strains *S. venezuelae ΔbldD*::apr, *S. venezuelae ΔbldN*::apr and *S. venezuelae ΔwhiB*::apr over 165 h. (**A**) Stereo microscope images of a single representative plaque for each strain taken at different time points after infection. (**B**) Plaque area (mm^2^) of 15 randomly chosen plaques per strain with calculated means shown in black (*S. venezuelae*), red (*ΔbldD*::apr), blue (*ΔbldN*::apr) and yellow (*ΔwhiB*::apr). (**C**) Decrease of plaque area in mm^2^ for each strain. Asterisks indicating significant differences (adjusted *P*-value < 0.001). Statistical analysis was performed as one-way ANOVA with Tukey's multiple comparison test in GraphPad Prism.

In order to observe the developmental changes during phage infection, we set up a time series experiment and monitored plaque growth of *S. venezuelae* NRRL B-65442 wild type as well as mutants defective at different stages of development using stereo microscopy (Fig. 2). In the case of the wild-type strain, maximal plaque size was observed approximately 42 h post infection (1.78 mm^2^ ± 0.51 mm^2^). From 42 hours onwards, plaques were shrinking in size (48.3% reduced plaque area (± 7.1%)) suggesting the regrowth of transiently phage resistant mycelium followed by the erection of aerial hyphae (∼66 h onwards; Fig. 2, Video S1).

To further assess the impact of cellular development on the containment of phage infection, we analyzed different *S. venezuelae* mutant strains defective at different stages of the development. These development mutants comprised the hypersporulating *S. venezuelae* strain ∆*bldD:: apr* (Tschowri et al. 2014) and strain Δ*bldN:: apr*, which is restricted to vegetative growth (Bibb et al. 2012). Further, we included the sporulation deficient strain *∆whiB:: apr*, which is still capable of the formation of aerial hyphae, but is blocked in the formation of spores (Bush et al. 2016). On this strain, phage Alderaan formed significantly smaller plaques (0.98 mm^2^ ± 0.49 mm^2^), likely caused by accelerated formation of aerial hyphae at the plaque interface in comparison to the wild type. However, also strain Δ*whiB* showed significant regrowth into the lysis zone (55.7% ± 11.0%)—at even earlier time points. However, reduction in plaque area was slightly less pronounced (Fig. 2C). Overexpressing *whiB* led to an abolishment of the growing and shrinking and yielded consistently smaller plaques (Fig. S1). Remarkably, strain Δ*bldN*, which is restricted to vegetative growth, featured large, clear plaques (1.43 mm^2^ ± 0.34 mm^2^) and no significant reduction in plaque area (19.0% ± 11.9%) (Fig. 2C). In contrast, Alderaan formed significantly smaller plaques on the hypersporulating strain Δ*bldD* (maximum 0.97 mm^2^ ± 0.24 mm^2^). At later time points, plaques became turbid (zones of regrowth), but did not show significant regrowth from the periphery of the plaque into the lysis zone (Fig. 2A). This is in agreement with the reported phenotype of this mutant, which displays an accelerated formation of spores largely bypassing the formation of aerial hyphae (Tschowri et al. 2014). Complementation of *bldD* (strain SVNT11) restored the differentiation surrounding the plaque and the growing and shrinking characteristic comparable to the WT as well as it yielded clear plaques again (Fig. S1).

Based on this comparative analysis of mutants defective at different stages of development, it was tempting to speculate that the emergence of transiently phage-resistant mycelium would allow regrowth into the lysis zone. To examine this premise, we sampled mycelium from the interface at different time points post infection. Infection with phage Alderaan of freshly inoculated cultures confirmed susceptibility to phage infection and is in line with the hypothesis of the formation of transiently phage-resistant mycelium at the infection interface (Fig. S2). Overall, these results indicate a critical role of *Streptomyces* development in containing phage infection and suggest that transition from vegetative mycelium to aerial hyphae (preserved in the wild-type and Δ*whiB* strain) as critical step.

**Figure 3.**
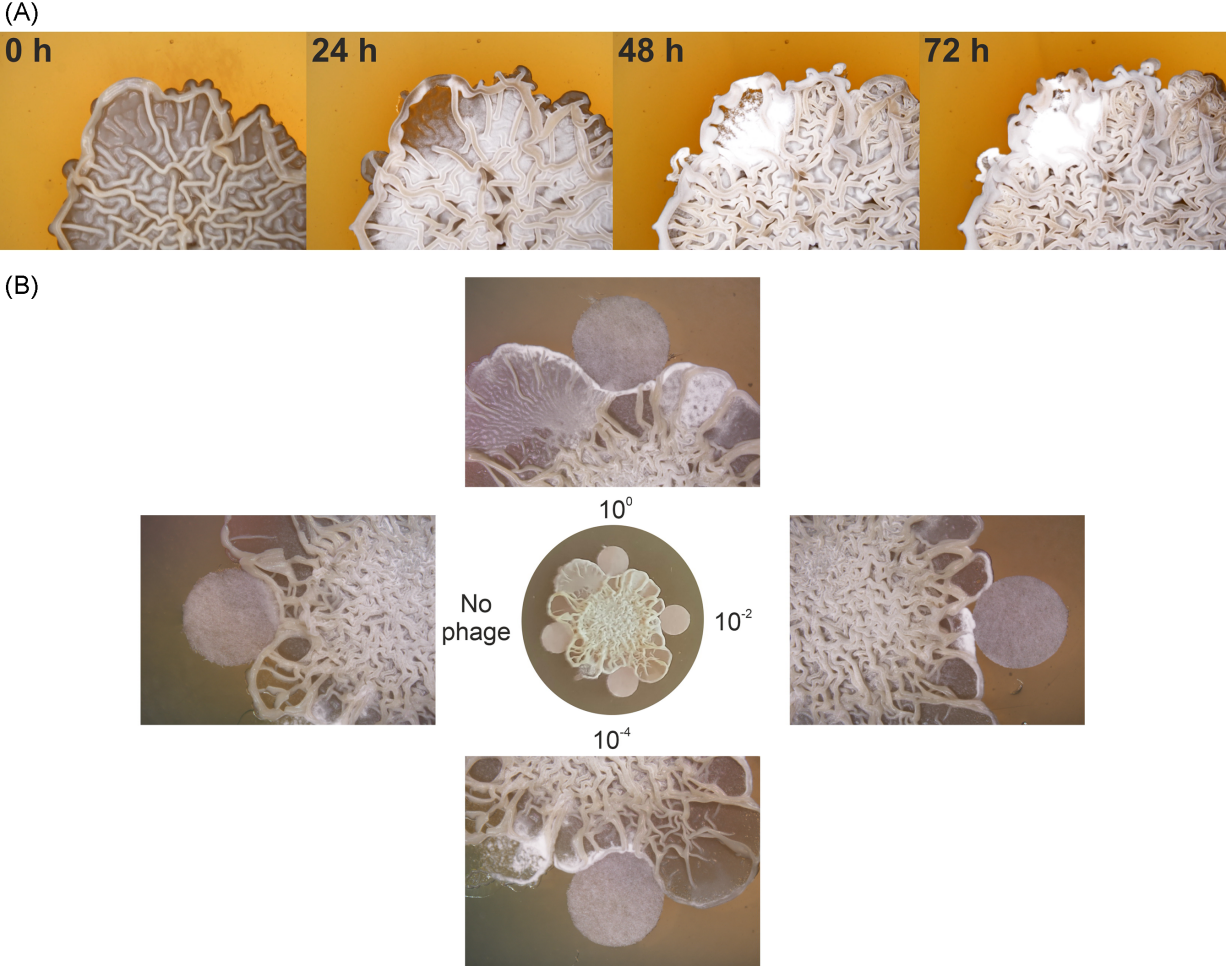
Sporulation of explorer cells in reaction to phage infection. (**A**) Stereo microscopy imaging of*S. venezuelae* explorer cells infected with phage Alderaan (after 6 days of exploration). (**B**) Explorer cells were imaged 3 days after contact with phage-soaked filter disks at indicated titers or soaked with phage buffer as control (no phage).

Besides the classical development cycle, *S. venezuelae* can enter another type of growth referred to as the exploration phenotype. Exploration is characterized by the rapid spreading of explorer mycelium across surfaces and is triggered by glucose starvation or in the presence of fungal competitors (Jones et al. 2017). Yet the impact of phage predation on exploratory growth is unknown. Spotting of phage Alderaan onto explorer cells caused a clearance of the region followed by regrowth and enhanced sporulation (Fig. 3A, Video S2). Similarly, explorer cells developed into spores when encountering paper disks soaked with phages (Fig. 3B). This suggests that sporulation is a key strategy to contain phage outbreaks in both the canonical and exploratory lifestyles.

### Fluorescent microscopy visualizes developmental patterns emerging upon phage infection

**Figure 4.**
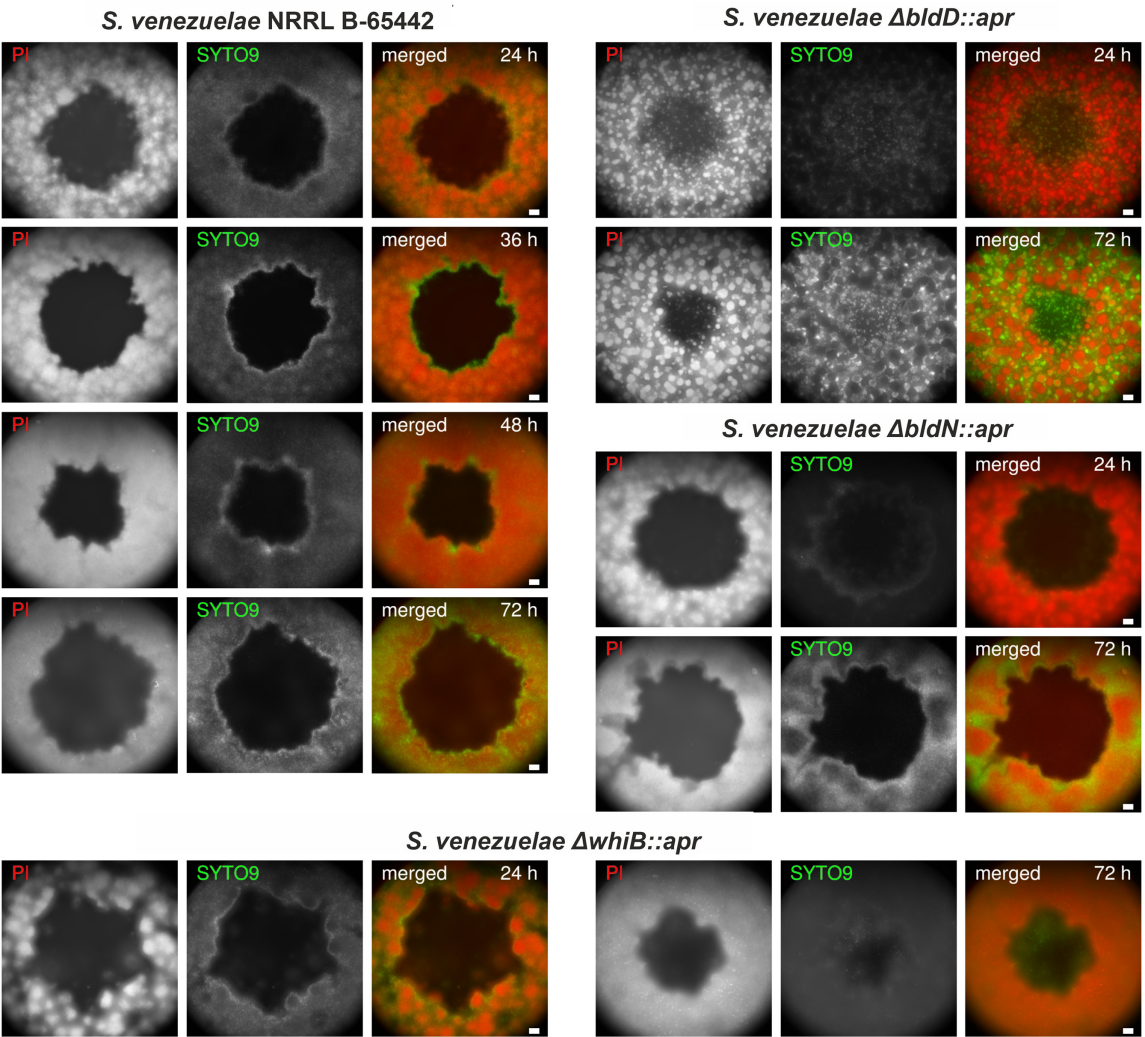
Analysis of Alderaan plaques on *S. venezuelae* indicates distinct developmental pattern formation in response to phage infection. Wide-field fluorescence microscopy of stained plaques and surrounding tissue of *S. venezuelae* wild type (WT) and different development mutants (*ΔbldD, ΔbldN* and *ΔwhiB*). Representative images of three biological replicates are shown. Cells were stained with propidium iodide (PI, red) and SYTO9 (green). Both channels are shown separately in grey scales and as a merged image. Scale bars represent 100 μm.

The analysis of plaque formation of phage Alderaan on *S. venezuelae* wild type and different development mutants revealed enhanced formation of aerial hyphae and spores at the plaque interface. To further analyze the spatiotemporal occurrence of developmental patterns post phage infection, we performed staining of single plaques and the surrounding tissue using the fluorescent dyes SYTO9 (green, stains nucleic acids in all cells) and propidium iodide (red, stains nucleic acids in cells with enhanced permeability). This stain is typically referred to as ‘live/dead’ staining, but it has to be kept in mind that the combination of these two stains allows for the visualization of differences in membrane permeability and membrane potential rather than for a strict discrimination between live and dead cells (Kirchhoff and Cypionka 2017). In the case of *S. venezuelae* wild type infected with Alderaan, a green ring emerged at the plaque interface after 36 h, indicative of differences in cellular development (Fig. 4). This observation is in line with the observed plaque shrinkage linked to the formation of aerial hyphae and spores at the infection interface (Figs. 1 and 2) and is further supported by previous studies showing that *Streptomyces* spores are impermeable to PI staining (Ladwig et al. 2015). This overall distinct pattern was not observed for the hypersporulating strain Δ*bldD* or the vegetatively growing strain Δ*bldN*. However, a faint pattern was also observed for the ∆*whiB* strain after 24 hours showing a higher level of SYTO9 staining at the plaque interface. This fits to the above-described observation that the ∆*whiB* strain featured an accelerated formation of aerial hyphae and a reduction in plaque area over the course of infection (Fig. 2C). Interestingly, *S. venezuelae* ∆*bldD* expressed complementary patches of PI and SYTO9 stained tissue in the lawn, but this pattern formation appeared to be independent of phage infection. Within the lysis zone of the plaque, we observed clusters of SYTO9 stained cells indicating faster regrowth of the hypersporulating ∆*bldD* strain in comparison to the wild type, which is in agreement with the formation of turbid plaques (Fig. 2A).

### Sporulation fosters the emergence of phage resistance

**Figure 5.**
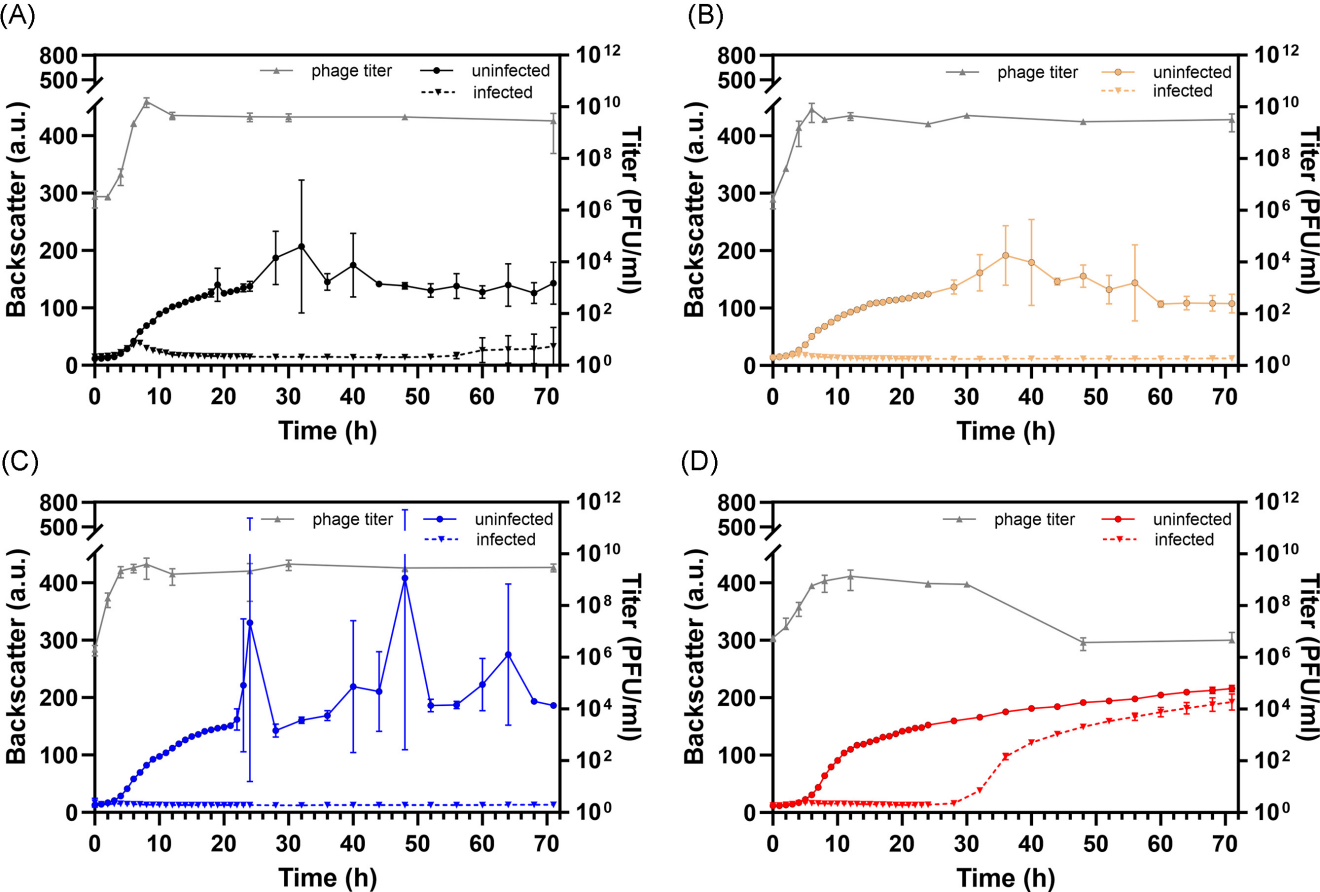
Enhanced sporulation of *S. venezuelae ΔbldD* enables the faster regrowth of phage-resistant cells. Infection of *S. venezuelae* WT (**A**) and different development mutants *S. venezuelae ΔwhiB*::apr (**B**), *ΔbldN*::apr (**C**) and *ΔbldD*::apr (**D**) with Alderaan over 72 h in GYM medium. Strains were cultivated in 48-well microtiter plates (see material and methods) and infection was started with an initial titer of 10^8^ PFU/ml (n = 3). Phage titer (PFU/ml, grey line+triangles) was determined by agar overlay assays (n = 3). An additional infection experiment with an initial titer of 10^7^ PFU/ml is shown in Fig. S4.

Analysis of Alderaan plaques revealed faster regrowth of the hypersporulating strain *S. venezuelae* Δ*bldD* within the plaque in comparison to wild-type lawns. Since *S. venezuelae* is able to complete its developmental cycle also in liquid culture, we set to examine whether infection of *S. venezuelae* with phage Alderaan in liquid cultures would follow a similar pattern. Infection at an initial titer of 10^8^ PFU/ml resulted in a culture collapse of the wild type and all three developmentally impaired strains compared to uninfected controls (Fig. 5). Over the course of the experiment, pH values stayed constant between 7.0 and 7.5 for all strains, infected and uninfected. Interestingly, the hypersporulating strain Δ*bldD* showed reproducibly faster regrowth after 30–40 h upon phage infection (Fig. 5D). Regrowth was also observed for the wild-type strain in several experiments but at later time points and with a high variability in timing. Reinfection of strains recovered from the Δ*bldD* culture after 70 h (Fig. 5) revealed complete resistance to infection with phage Alderaan on plates (Fig. S3). Measurement of the phage titer over time showed the maximal titer at approximately 8 h post infection. However, regrowth of Δ*bldD* coincided with a significant drop in phage titer (Fig. 5D). Notably, using a lower initial phage titer of 10^7^ PFU/ml resulted in an only very minor growth defect of the wild type but also showed faster regrowth of the Δ*bldD* strain in comparison to Δ*bldN* and *ΔwhiB* (Fig. S4). These data suggested an overall lower susceptibility of the wild type, in particular evident at low phage titers.

In conclusion, enhanced sporulation seems to favour regrowth following culture collapse in liquid cultures. In accordance with the findings reported above, sporulation appears to be associated with decreased phage susceptibility and supports population expansion under phage challenge in *Streptomyces*.

### Mature mycelium is less susceptible to phage infection

**Figure 6.**
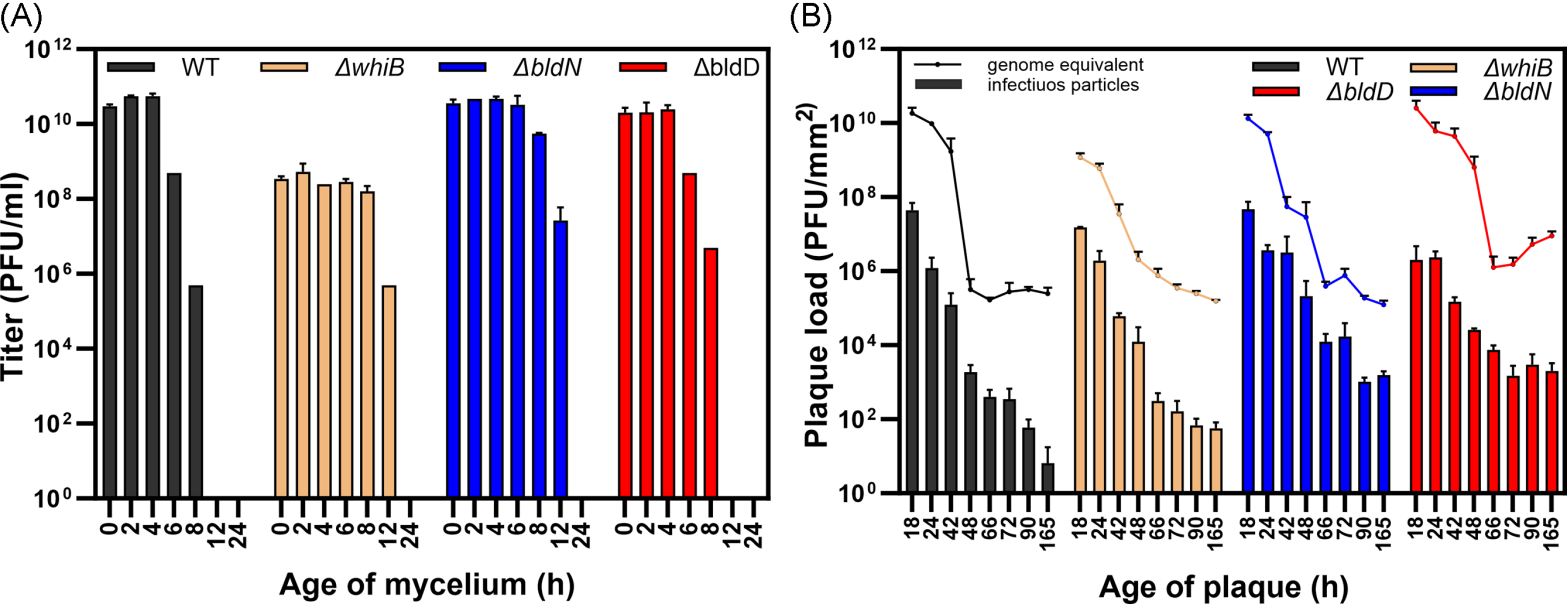
Mature mycelium is less susceptible to phage infection. (**A**) Spotting of phage Alderaan on lawns of *S. venezuelae* strains at different time points after plating (n = 2). (**B**) Determination of plaque load and infectivity of Alderaan particles from plaques as genome equivalents quantified by qPCR (line plot, n = 3) and infectious particles quantified by double agar overlay (bar plot, n = 3) in PFU/mm^2^. See Fig. S5 for details on plaque morphology.

Regrowth of the plaque periphery (Fig. 2) and the emergence of aerial hyphae and sporulation at the plaque interface suggested an impact of the developmental stage on phage susceptibility. We further addressed this hypothesis by spotting phage Alderaan on *S. venezuelae* mycelium grown on agar overlay plates (0–24 h; Fig. 6A). Remarkably, all four strains were resistant to infections when grown for 24 h prior to spotting. At this stage, a mature and dense vegetative mycelium seems to significantly reduce phage susceptibility. Interestingly, a similar phenotype was already observed at 8 and 12 h post incubation in the case of *S. venezuelae* wild type and *∆bldD*, but was delayed for ∆*bldN* and ∆*whiB*. Besides the significant reduction in phage titer, plaque sizes were smaller at later time points, which is probably the result of less susceptible host cells and constrained diffusion of phage particles (Fig. S5). Additionally, phage adsorption to non-growing spores in SM buffer and old mycelium grown for 24 h in liquid culture was impaired when compared to freshly germinating spores in medium (Fig. S6).

These results indicated an important impact of *Streptomyces* development on the emergence of phage-resistant phenotypes. However, quantification of the plaque phage load by qPCR (genome equivalents of Alderaan) and measurement of infectivity (PFU/mm^2^) revealed also a significant reduction over time (Fig. 6B). This effect was most pronounced for the *S. venezuelae* wild-type strain showing a significant decrease in plaque load between 42 and 66 h. Taken together, these results emphasize the observed dynamics in plaque growth and shrinkage as a consequence of a reduced plaque load and the development of transiently phage-resistant mycelium.

### Phage infection influences expression of developmental genes and biosynthetic gene clusters

**Figure 7.**
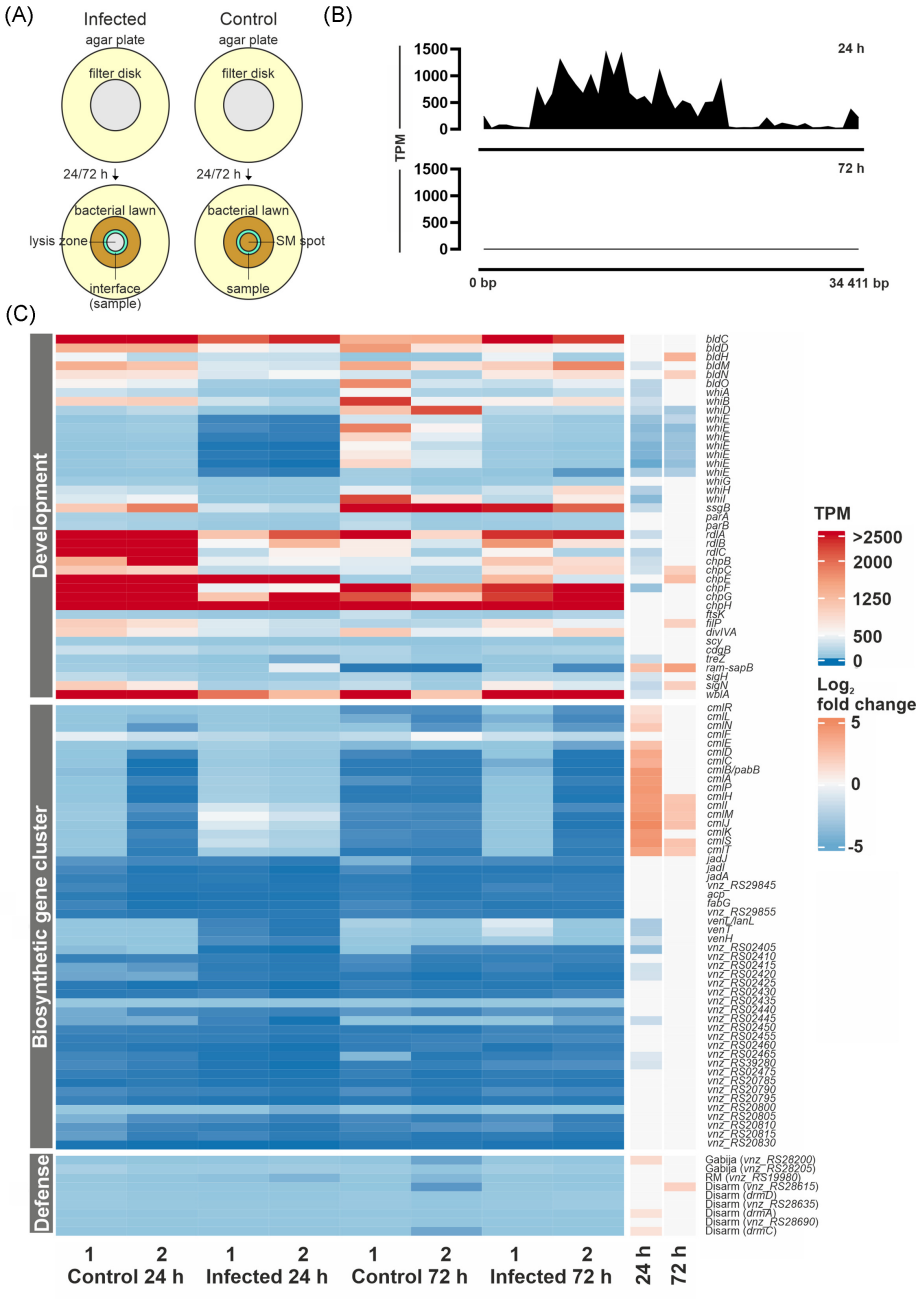
Global transcriptome analysis of *S. venezuelae* cells at the infection interface. (**A**) Sampling strategy of *S. venezuelae* lawns grown on filter disks and infected with Alderaan or spotted with SM buffer as control. Turquoise ring represents the sampling zone of cells at the infection interface, between lysis zone (grey) and bacterial lawn (brown). (**B**) Alderaan gene expression in transcripts per million (TPM) detected at different time points. (**C**) Heat map of selected genes involved in development, biosynthetic gene clusters and phage defense. Shown are expression levels in TPM ranging from 0 TPM (dark blue) to >2500 TPM (dark red) and data for two independent biological replicates (‘control’ or ‘infected’, 24 or 72 h). The log_2_ fold change calculated for infected vs control at the given time point is shown on the right, color-coded according to the legend ranging from blue (-5) to red (5). Empty boxes represent samples where differential gene expression was not significant (FDR *P*-value > 0.05).

To gain further insights into the processes underlying adaptation to phage infection, we performed an RNA-seq analysis of infected mycelium from the plaque interface of the *S. venezuelae* wild type harvested 24 and 72 h after infection with Alderaan (Fig. 7A). A comprehensive overview of the obtained data for all *S. venezuelae* and Alderaan genes can be found in Supplementary Table S5. While we observed high levels of Alderaan gene expression at the 24 h time point, infection appeared to be fully contained at 72 h showing almost no detectable expression of Alderaan genes (Fig. 7B). In addition, the integrated prophage Chymera (KU958700.1) ranging from 3114820 to 3149561 bp of the *S. venezuelae* genome shows several differentially expressed genes including for example the site-specific integrase. However, general expression levels are low indicating no complete prophage induction (Fig. S7).

Of the more than 7000 *S. venezuelae* genes, 2483 and 1305 genes were significantly up- or downregulated during phage infection after 24 and 72 h, respectively (FDR *P*-value ≤ 0.05; Table S5, Sheet 2 and 3). At 24 h the most upregulated gene during infection of *S. venezuelae* encodes a lysine tRNA (log_2_-fold change 7.7). Interestingly, there are 26 additional tRNA genes upregulated at this time ranging from a log_2_-fold change of 4.5 to 1.3. This trend abolished at the later 72 h time point when phage infection was fully contained.

At the early stages of infection, several genes involved in *Streptomyces* development showed strong downregulation (log_2_-fold change between -1.2 and -5.4), including almost all *whi* genes, *bldM* and *bldO, ssgB, treZ, sigH* and *sigN, rdlA* and *rdlC* as well as three of the six *chaplin* genes (*chpBCF*) (Fig. 7C; Table S5, Sheet 4). This repression of genes involved in cellular development coincided with the high expression of phage genes observed at the 24 h time point. After 72 h, when cellular development was visible at the infection interface, *whi* genes were not differentially expressed anymore except for *whiD* and *whiE* genes still showing reduced expression levels. In contrast, several genes involved in the formation of aerial hyphae showed enhanced expression, including *sapB, bldH, bldN, filP* and *sigN*. As targets of BldN, *chpC*, and *chpE* also featured increased expression at the infection interface after 72 h (Fig. 7C; Table S5, Sheet 4). These results provide important insights into the modulation of *Streptomyces* development during phage infection and suggest the repression of developmental processes during the early phase of infection. After 72 h, the containment of infection (no Alderaan transcripts), coincided with the enhanced expression of several genes involved in the formation of spore-bearing aerial hyphae.

Besides developmental processes affected by phage infection, genes from three of the five biosynthetic gene clusters in *S. venezuelae* featured differential expression during ongoing infection (24 h; Table S5, Sheet 5). Most importantly, 16 out of 17 genes from the chloramphenicol cluster are strongly upregulated during infection with Alderaan (Fig. 7C). Conversely, all three genes of the venezuelin gene cluster and six of 15 genes belonging to a cluster encoding five different compounds are downregulated (Table S5, Sheet 5). Jadomycin and gaburedin biosynthetic gene clusters are not influenced under the tested conditions. When testing for an effect of chloramphenicol on phage infection, Alderaan showed no decreased phage titer in the presence of different concentrations of the antibiotic (Fig. S8).

For detection of potential defense genes, DefenseFinder (https://defense-finder.mdmparis-lab.com/) (Abby et al. 2014, Tesson et al. 2022) and PADLOC (https://padloc.otago.ac.nz/padloc/) (Payne et al. 2021) were used to screen the *S. venezuelae* genome (NZ_CP018074) yielding a total of 19 genes belonging to eight different types of defense systems (Table S5, Sheet 6). Four of those genes belonging to three systems were detected by both tools (Fig. 7C). While all defense genes mostly have expression values in the low and middle two-digit range, except for *vnz_RS11150* encoding a dGTPase detected by PADLOC there is no system significantly and consistently up- or downregulated upon phage infection.

## Discussion

In this study, we demonstrated the important impact of cellular development on the emergence of transient phage resistance as part of the antiviral strategies employed by *Streptomyces*. Analysis of plaque formation—as the hallmark of phage infection—displayed that after a period of steep growth in plaque area, transiently phage-resistant mycelium reconquered the lysis zone. This process was dependent on the onset of cellular development, in particular the formation of aerial hyphae, as illustrated by the analysis of mutants defective at different stages of develop-ment.

The last few years have witnessed an unprecedented expansion in our knowledge on bacterial immune systems with the discovery of many new systems, some of which are conserved from bacteria to humans (Tal and Sorek 2022, Wein and Sorek 2022). Besides the innate and adaptive immune systems acting at the cellular level, multicellular antiviral strategies including the secretion of antiviral molecules, release of membrane vesicles and biofilm structure represent an emerging scheme in antiviral defense of bacteria (Kronheim et al. 2018, Bond et al. 2021, Hardy, Kever and Frunzke 2022, Kever et al. 2022). In this study, we showed that *S. venezuelae* is able to reconquer the lysis zone upon the late stages of phage infection. This development of aerial hyphae and spores at the plaque interface was, in fact, observed for a variety of different phages infecting *Streptomyces* species (Fig. 1). Mutational analysis revealed that the characteristic growth and constriction of the plaque is most pronounced in the *S. venezuelae* wild-type strain capable of full development. Interestingly, the observed phenotype is reminiscent of the plaque dynamics observed for phages SPP1 and Phi29 infecting *Bacillus subtilis*. There, the phenomenon of plaque constriction was attributed to a response modulated by the ECF sigma factor SigX leading to alterations of the modification of wall teichoic acids thereby interfering with phage attachment (Tzipilevich et al. 2022).

Further studies point towards the underappreciated prevalence of mechanisms involved in the generation of transient phage resistance. Those include the modification of capsule components in *Klebsiella* (Hesse et al. 2020), the O-antigen length in *Salmonella* (Cota et al. 2015), or the shedding of cell wall components, which was recently shown to render *Streptomyces, E. coli*, and *B. subtilis* transiently resistant to phage infection in osmoprotective environments (Fabijan et al. 2021, Ongenae, Briegel and Claessen 2021, Ongenae et al. 2022).

These studies add an additional layer of complexity to the general assumption that plaque growth is mainly constrained by the entry of bacterial cells into the stationary phase (Abedon and Yin 2009). When defining size and kinetics of a plaque, diffusivity, burst size, adsorption rate, and latent period are important phage-related parameters to consider (Abedon and Yin 2009, Gallet, Kannoly and Wang 2011, Abedon 2018). As shown in our study, Alderaan infecting *S. venezuelae* formed significantly smaller plaques on older mycelium probably due to reduced diffusivity in dense mycelial structures.

Differentiation from vegetative mycelium to aerial hyphae and spores also comes with changes in the cell surface including the formation of a hydrophobic rodlet structure (Claessen et al. 2004, Yang et al. 2017) as well as compositional changes and thickening of the spore envelope (Bradley and Ritzi 1968, Sexton and Tocheva 2020) thus influencing phage adsorption as shown here. This is underlined by the ability of fungal mycelium to retain phages and reduce the PFU as well as mass yields correlating with hydrophobicity (Ghanem et al. 2019). Studies on phage-host interactions in *E. coli* biofilms also conclude that reduced diffusivity and cell surface protection are key strategies in spatially constrained environments, although these phages can remain infectious and act as an additional layer of protection against incoming competitors (Vidakovic et al. 2018, Bond et al. 2021). In addition, spores being by definition metabolically inactive, productive phage infection can only occur upon germination as it was for example shown for endospores of *B. subtilis* where infection and lysis halted during the spore phase (Gabiatti et al. 2018).

Following a typical parasitic behavior, phages also manipulate cellular development to promote their own distribution through spores, as it was observed for example with the *B. subtilis* prophage SPβ regulating sporulation (Abe et al. 2014). This is further supported by the ubiquitous distribution of phage-encoded sporulation-specific sigma factors with a proposed role in altering bacterial dormancy (Schwartz, Lehmkuhl and Lennon 2022). In this study, transcriptome analysis revealed the repression of *S. venezuelae* developmental genes in the early stages of active Alderaan infection suggesting phage-driven measures enhancing propagation. While sporulation-specific sigma factors are ubiquitously found in Firmicutes, WhiB-like transcriptional regulators are prevalent in the genomes of actinobacteriophages including phage Alderaan (Sharma et al. 2021). WhiB-like regulators have been shown by various studies to play diverse roles in controlling cellular development, oxidative stress response, production of secondary metabolites and cell division (Bush 2018). It is therefore tempting to assume that phages infecting bacteria undergoing cellular development are equipped with diverse measures to modulate development promoting phage propagation and distribution.

Recent studies revealed that secondary metabolites of the class of anthracyclines and aminoglycoside antibiotics, produced by *Streptomyces*, provide protection against phage infection (Kronheim et al. 2018, Hardy, Kever and Frunzke 2022, Kever et al. 2022). Besides first phenomenological evidence for the phage-triggered induction of actinorhodin production (Hardy et al. 2020), the effect of phage infection on the activation of biosynthetic gene clusters has not yet been investigated. In our study, transcriptome analysis revealed an induction of the chloramphenicol gene cluster upon Alderaan infection. A direct antiphage effect of chloramphenicol was not observed under the tested lab conditions. However, interaction in natural microbial communities are multi-layered and the production of antibiotics affecting translation might suppress immune adaptation of host populations (Chevallereau et al. 2020). In this context, a recent study provides evidence for a role of antibiotics inhibiting protein synthesis in the prevention of phage amplification by interfering with the production of counter-defences, such as the synthesis of anti-CRISPR proteins (Pons et al. 2022).

In conclusion, our study emphasizes the importance of cellular development for the emergence of transient phage resistance during multicellular development of *Streptomyces*. These results suggest that phage infection in multicellular bacterial assemblies is sensed and triggers the establishment of a resistance response—here the formation of aerial hyphae and spores—with an important role for the containment of infections. It remains subject of further investigations, whether this transient resistance might accelerate the acquisition of genetic resistance towards phage infection, as described for phenotypic tolerance to antibiotics facilitating the evolution of resistance (Liu et al. 2020).

## Material and methods

### Bacteria, phages and growth conditions

Tables S1 and S2 list all bacteriophages and bacterial strains used in this study, respectively. For liquid cultivation of wild type strains, spore stocks were used for inoculation whereas developmental mutant strains were inoculated from mycelial stocks. Cultivation was performed in GYM medium (per liter: 4 g glucose, 4 g yeast extract, 10 g malt extract, pH = 7.3) at 30°C and 170 rpm. *S. coelicolor* was cultivated in YEME medium (per liter: 3 g yeast extract, 3 g malt extract, 5 g peptone, 10 g glucose, 340 g sucrose). For growth of mutant strains in the absence of phages, apramycin was added to precultures at a concentration of 10 µg/ml. For solid growth medium, GYM agar was used for all strains and experiments (GYM medium plus 2 g CaCO_3_ per liter, 1.5% agar). Triggering exploration phenotypes was achieved by using YP agar (per liter: 10 g yeast extract, 20 g peptone, 1.5% agar) supplemented with 10 mM MgCl_2_ and 10 mM CaCl_2_ to support phage amplification. *Escherichia coli* strains DH5α and ET12567/pUZ8002 were cultivated in LB medium supplemented with the respective antibiotics during cloning.

### Plasmid construction and cloning

Details concerning plasmids and oligonucleotides used for constructing plasmids pIJ10257_*whiB_Sven_* and pSVNT-3 are listed in Tables S3 and S4, respectively. PCR amplification and restriction digestion were performed as described in standard protocols (Sambrook and Russel 2001). Plasmid construction was done using Gibson assembly (Gibson 2011). Synthesis of oligonucleotides and sequencing were performed by Eurofins Genomics (Ebersberg, Germany). Transfer of plasmids into *Streptomyces spp*. was done using conjugation through the *E. coli* ET12567/pUZ8002 strain (MacNeil et al. 1992).

### Phage infection on solid media

Double agar overlays were performed using GYM soft agar without CaCO_3_ and only 0.4% agar for the top layer. Quantification of infectious phage particles was performed by spotting 2 µl of phage dilutions in SM buffer (0.1 M NaCl, 8 mM MgSO_4_, 50 mM Tris-HCl, pH 7.5) onto a double agar overlay inoculated with the respective *Streptomyces* species at an initial OD_450_ of 0.4 from overnight cultures or directly mixing 100 µl of the phage solution into the inoculated soft agar before plating.

For re-infecting experiments, mycelium was harvested after different time points from plaque interface. Triplicates from individual plaques per strain at each time point were picked using a sterile loop and incubated for 24 h at 30°C and 900 rpm in 1 ml of fresh medium. About 250 µl of these cultures were used for a double agar overlay and spotting of phage dilutions.

Testing of phage susceptibility to chloramphenicol was performed as described previously (Kever et al. 2022). In this case, GYM agar for bottom and top layers were supplemented with 0, 10, 50, 100, or 200 µg/ml chloramphenicol when used for spotting of Alderaan dilutions.

### Infection in liquid cultures

Infection of liquid cultures was performed in the BioLector microcultivation system (Beckman Coulter, US) (Kensy et al. 2009). Overnight cultures were adjusted to an OD_450_ of 0.15 and centrifuged for 5 min at 5000 x *g*, the supernatant was discarded and the pellet resuspended in 1/10 of the initial volume. Phages were added to a final titer of 10^8^ PFU/ml and SM buffer was used for uninfected controls. After 10 minutes of incubation at room temperature, free phages were removed by centrifugation for 5 min at 5000 × *g* and discarding the supernatant. The pellet was resuspended in the initial volume of GYM medium. For cultivation, biological triplicates were incubated at 30°C and 1200 rpm in FlowerPlates (Beckman Coulter, US). The backscattered light intensity was measured in 15 minutes intervals with a wavelength of 620 nm (gain of 25). For determination of phage titer over the course of infection, supernatants were sampled at indicated time points and subsequently spotted on double agar overlays as described above.

### Imaging and measuring of plaques

Stereo microscopy was performed using the SMZ-18 stereomicroscope (Nikon, Japan) equipped with a P2-SHR Plan Apo 1x N.A. objective (Nikon, Japan), a P2-DBL LED plain base (Nikon, Japan) and a DS-Fi3 digital microscope camera (Nikon, Japan). The H301-MINI temperature-controlled chamber (Okolab, Italy) and UNO-STAGE-TOP-INCUBATOR (Okolab, Italy) were used for incubation at 30°C during live-imaging. Image and video processing and measuring of plaque sizes was done using the NIS-Elements AR v.5.30.03 (Nikon, Japan).

### Electron microscopy

To visualize the mycelial differentiation at the infection interface in comparison to the uninfected lawn, double agar overlays were conducted as described in ‘Phage infection on solid media’. After 72 h post infection, agar plates were overlaid with 4 ml B2 buffer (87 mM Na_2_HPO_4_×2H_2_O, 13 mM NaH_2_PO_4_ x H_2_O, pH 7.4) containing 3% glutaraldehyde and incubated for 1 h at RT, before storing at 4°C until imaging. Microscopic analysis was conducted in the facility for electron microscopy (EME, institute for pathology) at the Universitätsklinikum Aachen (Germany).

### Fluorescence microscopy of plaques

After infection on solid medium three single plaques were randomly selected for each strain and time point and were cut out of the agar as small blocks. Neutral buffered formalin 10% (v/v) in 1x PBS (137 mM NaCl, 2.7 mM KCl, 10 mM Na_2_HPO_4_, 2 mM K_2_HPO_4_) was used for fixation of the plaques by incubation for 12–18 h. Afterwards plaques were washed three times in 1x PBS and stored at 4°C until microscopy. Live-dead staining was performed directly before imaging with 5 µl 1:2 dilution of the staining mix containing propidium iodide and SYTO9 (LIVE/DEAD™ *Bac*Light™ Bacterial Viability Kit, Invitrogen) placed directly onto the plaque and incubated for 5 min at room temperature in the dark.

Epifluorescence microscopy was applied at an inverted Nikon Eclipse Ti microscope system (Nikon instruments, Japan) equipped with a Nikon S Fluor 10x DIC N1 dry objective lens and a Hammamatsu Orca Flash 4.0 (Hammamatsu Photonics, Japan). Stained samples were imaged face down and half submerged in water in a 35 mm glass Bottom µ-Dish (Ibidi, Germany). To measure the SYTO™ compound of the BacLight™ stain, a Nikon GFPHQ Filterset (Ex 470/40 DM 495LP Em 525/50) was utilized with 30 ms exposure time and a Nikon INTENSILIGHT C-HGFIE as light source, while for the corresponding propidium iodide signal a Nikon TXRed HYQ filterset (Ex 560/55 DM 595LP Em 645/75) was used with 7 ms exposure time. Z-Stacks of the full sample were acquired for all channels at a step size of 1 µm. Subsequently, maximum intensity projections were generated within a local omero image database installation, which was used to generate figures of microscopic measurements as well.

### Plaque load determination

Quantification of phage particles per plaque over time was performed using qPCR and determination of PFU/mm^2^ using double agar ovelay assay as described above. Infection was done as described in ‘Phage infection on solid media’ on GYM agar. For each time point, three plaques were imaged and measured as described in ‘Imaging of plaques’ and cut out as small agar blocks. The plaques were submerged in SM buffer for 2 h at 500 rpm and room temperature before centrifugation for 20 min at 8000 x g. The supernatant was stored at 4°C until further use. For qPCR, 1 µl of the supernatant was mixed with 10 µl 2x Universal qPCR Master Mix (New England Biolabs, USA) and 1 µl of each primer targeting the minor tail gene ( Alderaan_HQ601_00028_fw: CTCGGCTATCCGATCATCC; Alderaan_HQ601_00028_rev: TTGGTTGCGGTTGATGGAC; final conc. 0.5 µM) in a final volume of 20 µl with dd. H_2_O. Reactions were conducted in the qTower 2.2 (Analytik Jena, Germany) in duplicates with phage lysate dilutions as standards. The resulting concentrations were used to calculate the plaque load as genome equivalents in PFU/mm^2^ using the initial volume of SM buffer and the measured plaque size.

Quantification of plaque load as infectious particles in PFU/mm^2^ was performed as described in ‘Phage infection on solid medium’ on *S. venezuelae* NRRL B-65442 with the herein supernatant as input phage solution.

### Transcriptome analysis

For transcriptomic analyses of *S. venezuelae* NRRL B-65442, 1 ml of an overnight culture adjusted to an OD_450_ of 2 was transferred onto a nylon membrane filter (0.45 µm pore size, diameter 47 mm, Cytiva, USA) placed on a GYM agar plate. After air-drying, 10 µl Alderaan phage stock (10^10^ PFU/ml) or SM buffer as control were spotted in the middle of the filter. Incubation was performed at 30°C for 24 h or 72 h. Mycelial material from the edge of formed spots was scraped from the filter and ground up under liquid nitrogen. RNA purification was done using the Monarch Total RNA Miniprep Kit (New England Biolabs, USA). Depletion of rRNA, library preparation and sequencing were conducted by GENEWIZ (Germany).

Analysis of sequencing results was performed using CLC genomics workbench v.20 (Qiagen, Germany). First, quality control of the reads was conducted and subsequent trimming for adapter sequences and low-quality reads (0.05) as well as ambiguous nucleotides was performed. As references, *S. venezuelae* NRRL B-65442 (NZ_CP018074.1) and Alderaan (MT711975.1) genomes were used for read mapping. Transcripts per million (TPM) were calculated with the ‘RNA-seq Analysis’ tool of CLC genomics workbench (mismatch cost 2; insertion cost 3; deletion cost 3; length fraction 0.9; similarity fraction 0.9; strand specificity both; maximum number of hits for a read 10). Determination of differentially expressed genes was done using the ‘Differential Expression in Two Groups’ tool in CLC combining duplicates for comparing infected and uninfected samples at 24 and 72 h separately with an FDR *P*-value of ≤ 0.05. A heatmap presenting the TPM annotated with differential expression values was constructed in R using the ComplexHeatmap package (Gu, Eils and Schlesner 2016).

## Supplementary Material

uqad002_Supplemental_Files

## Data Availability

The RNA-sequencing data discussed in this publication have been deposited in NCBI's Gene Expression Omnibus (Edgar, Domrachev and Lash 2002) under the accession number GSE213718.
