# Supplementary material for: Streptomyces development is involved in the efficient containment of viral infections
Source: Microlife. 2023 Jan 16;4:uqad002. doi: 10.1093/femsml/uqad002 (PMC10117723; doi:10.1093/femsml/uqad002)
Supplement: uqad002_Supplemental_Files [file uqad002_supplemental_files.zip › 230109_Luthe et al SI.docx]

**Supplementary Information to**

***Streptomyces* development is involved in the efficient containment of viral infections**

Tom Luthe^1^, Larissa Kever^1^, Sebastian Hänsch^2^, Aël Hardy^1^, Natalia Tschowri^3^ Stefanie Weidtkamp-Peters^2^, Julia Frunzke^1*^

^1^Institute of Bio- and Geosciences, IBG-1: Biotechnology, Forschungszentrum Jülich, 52425 Jülich, Germany

^2^Center for Advanced Imaging, Heinrich Heine Universität Düsseldorf, 40225 Düsseldorf, Germany

^3^Institute of Microbiology, Leibniz Universität Hannover, 30419 Hannover, Germany

*Corresponding author:

Julia Frunzke; Email: [j.frunzke@fz-juelich.de](mailto:j.frunzke@fz-juelich.de), Phone: +49 2461 615430

**Tables**

**Supplementary Table S1: Phages used in this study.**

**Supplementary Table S2: Bacterial strains used in this study.**

**Supplementary Table S3: Plasmids used in this study.**

**Supplementary Table S4: Oligonucleotides used in this study.**

**Supplementary Table S5: RNA-Sequencing Data (separate file).** For transcriptome analysis of Alderaan infected mycelium from *S. venezuelae*, plaque interface samples were taken as described in Figure 7A and subsequently processed and analyzed as described in the material and methods section. Here we present a complete overview of the expression of Alderaan and *S.venezuelae* genes as transcripts per million from two replicates for each time point under infected and uninfected control conditions (Sheet 1). Additionally, we show the significantly (p ≤ 0.05) differentially expressed genes as log2 fold changes at 24 h (Sheet 2) as well as 72 h (Sheet 3) after infection and from that filter developmental genes (Sheet 4), biosynthetic gene clusters (Sheet 5) and defense genes (Sheet 6).

**Figures**

**Supplementary Figure S1: Plaque development on the *whiB* overexpressing strain and the *bldD* complementation SVNT11.**

**Supplementary Figure S2: Re-infection of mycelium from plaque interfaces.**

**Supplementary Figure S3: Plaque count of Alderaan on regrown *ΔbldD* mycelium from liquid infections.**

**Supplementary Figure S4: Liquid infection of *S. venezuelae* strains with Alderaan.**

**Supplementary Figure S5: Plaques of Alderaan on aged mycelium of *S. venezuelae*.**

**Supplementary Figure S6: Adsorption of phage Alderaan to *S. venezuelae*.**

**Supplementary Figure S7: Expression of the Chymera genome during infection of *S. venezuelae*.**

**Supplementary Figure S8: Plaque formation of Alderaan in presence of chloramphenicol.**

**Videos**

**Supplementary Video S1: Dynamics of plaque growth and constriction of *S. venezuelae* infected with phage Alderaan (separate file).** A double agar overlay of phage Alderaan on *S. venezuelae* was performed on GYM agar and incubated for 18 h at 30 °C before live-imaging a single plaque for additional 72 h every hour under the stereomicroscope.

**Supplementary Video S2: Infection of *S. venezuelae* explorer cells by phage Alderaan (separate file).** *S. venezuelae* was grown for 6 days at 30 °C on YP agar supplemented with 10 mM MgCl_2_ and 10 mM CaCl_2_ before 4 µl of a 10^9^ PFU/ml Alderaan solution were spotted on one edge of exploring cells. Live-images were taken under the stereomicroscope every 30 min for 65 h.

**Tables**

**Supplementary Table S1: Phages used in this study.**

| Phage | Host organism | Taxonomy | Cluster (phagesdb.org) | Lifestyle | Reference |
| --- | --- | --- | --- | --- | --- |
| Alderaan | *S. venezuelae*NRRL B-65442 | *Austintatiousvirus* | BC | Temperate^1,2^ / Virulent^3,4^ | (Hardy *et al.* 2020) |
| SV1 | *S. venezuelae*NRRL B-65442 | *Picardvirus* | BC | Temperate^1^ / Virulent^3,5^ | (Stuttard 1979) |
| phi A.streptomycini III | *S. griseus* DSM 40236 | *Woodruffvirus* | BG | Virulent^1,3,4^ | DSM 49153 |
| P26 | *S. griseus* DSM 40236 | *Woodruffvirus* | BG | Virulent^1,3,4^ | DSM 49026 |
| Dagobah | *S. coelicolor* M145 | Viruses | Singleton | Temperate^2,3,5^ | (Hardy *et al.* 2020) |
| Endor1 | *S. coelicolor* M145 | *Camvirus* | BD | Temperate^1,2,3,5^ | (Hardy *et al.* 2020) |

^1^according to cluster; ^2^according to PhageAI; ^3^according to plaque morphology; ^4^according to absence of integrase and lysogens; ^5^according to literature

**Supplementary Table S2: Bacterial strains used in this study.**

| Strain | Genotype | Reference |
| --- | --- | --- |
| *Streptomyces venezuelae*  NRRL B-65442 | Wild-type strain | (Gomez-Escribano *et al.* 2021) |
| *S. venezuelae ∆bldD::apr* | *∆bldD::apr* | (Tschowri *et al.* 2014) |
| *S. venezuelae ∆bldN::apr* | *∆bldN::apr* | (Bibb *et al.* 2012) |
| *S. venezuelae ∆whiB::apr* | *∆whiB::apr* | (Bush *et al.* 2016) |
| *S. griseus* DSM 40236 | Wild-type strain | (Liu *et al.* 2005) |
| *S. coelicolor* M145 | *S. coelicolor* A3(2) lacking plasmids SCP1 and SCP2 | (Kieser *et al.* 2000) |
| *S. venezuelae* *whiB_Sven_* OE | *S. venezuelae* NRRL B-65442 carrying plasmid pIJ10257_*whiB_Sven_* | This study |
| *S. venezuelae* *∆bldD::apr /* pMS82*-bldD* (SVNT11) | *S. venezuelae ΔbldD::*apr carrying plasmid pSVNT-3 | This study |
| *Escherichia coli* DH5α | *supE44 ΔlacU169 (f80lacZDM15) hsdR17 recA1 endA1 gyrA96 thi-1 relA1* | Invitrogen |
| *Escherichia coli* ET12567/pUZ8002 | *dam-13∷Tn9 dcm-6 hsdM hsdR,* carrying plasmid pUZ8002 | (MacNeil *et al.* 1992) |

**Supplementary Table S3: Plasmids used in this study.**

| Plasmid | Characteristics | | | | | Reference | |
| --- | --- | --- | --- | --- | --- | --- | --- |
| pIJ10257 | Hyg^R^; constitutive ermE* promoter; ΦBT1 phage integration site | | | | | (Hong *et al.* 2005) | |
| pMS82 | Hyg^R^; ΦBT1 phage integration site | | | | | (Gregory, Till and Smith 2003) | |
| Plasmid | **Characteristics** | **Template** | **Primer** | **Vector** | **Restriction enzymes** | **Sequencing primer** | **Ref.** |
| pIJ10257_*whiB_Sven_* OE | *whiB* vnz_RS13740 of *S. venezuelae* | *S. venezuelae* NRRL B-65442 | 1 + 2 | pIJ10257 | HindIII;  NdeI | 3 + 4 | This study |
| pSVNT-3 | *bldD* vnz_RS05310  with 534 bp upstream and 404 bp downstream of the ORF | *S. venezuelae* NRRL B-65442 | 5 + 6 | pMS82 | HindIII;  KpnI | 7 + 8 | This study |

**Supplementary Table S4: Oligonucleotides used in this study.**

| Number | Name | Sequence |
| --- | --- | --- |
| 1 | Sven_whiB_pIJ10257_fwd | AGAACAGGAGGCCCCATATGATGACCGAGTTGTTCCAGGA |
| 2 | Sven_whiB_pIJ10257_rev | AGAACCTAGGATCCAAGCTTTCAGACGGCGGCCTT |
| 3 | pIJ10257_seq_fw | TGGCACCGCGATGCTGTTGT |
| 4 | pIJ10257_seq_rev | TCAGCGAGCTGAAGAAAGAC |
| 5 | bldD_HindIII_pMS82_long_fw | GTCAAGCTTGATCTCGGTGCGGCCGATG |
| 6 | bldD_KpnI_pMS82_long_rev | CCTGGGTACCGAGCTCATCGCGACCTACG |
| 7 | pMS82-seq-fw | GATGTCATCAGCGGTGGAGT |
| 8 | pMS82-seq-rev | CTGATGTGCTCAGTATCAC |

**Figures**


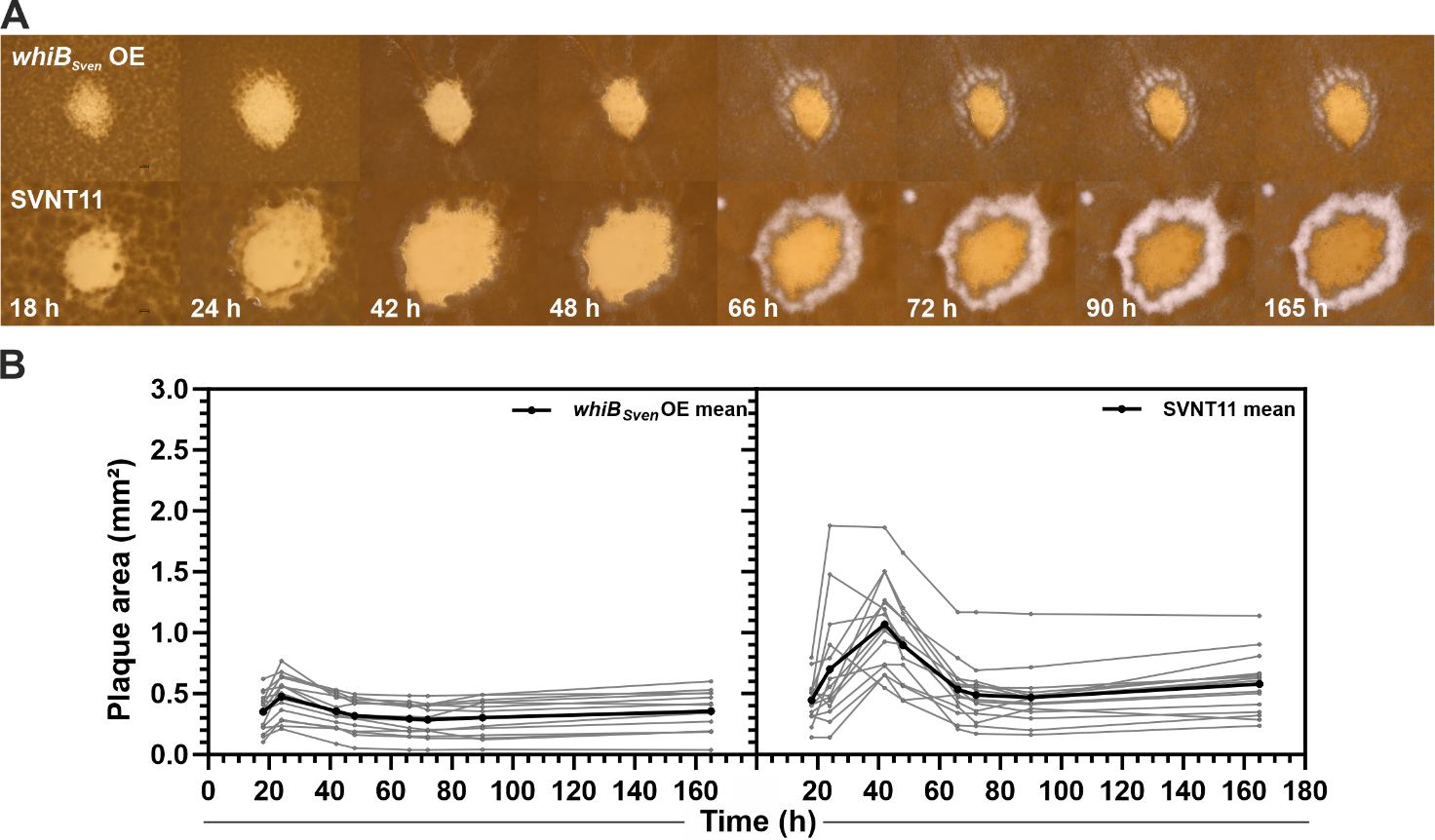


**Supplementary Figure S1: Plaque development on the whiB overexpressing strain and the bldD complementation SVNT11. (A)** Stereo microscope images of a single representative plaque for each strain taken at different time points after infection. **(B)** Plaque area (mm^2^) of 15 randomly chosen plaques per strain with calculated means.


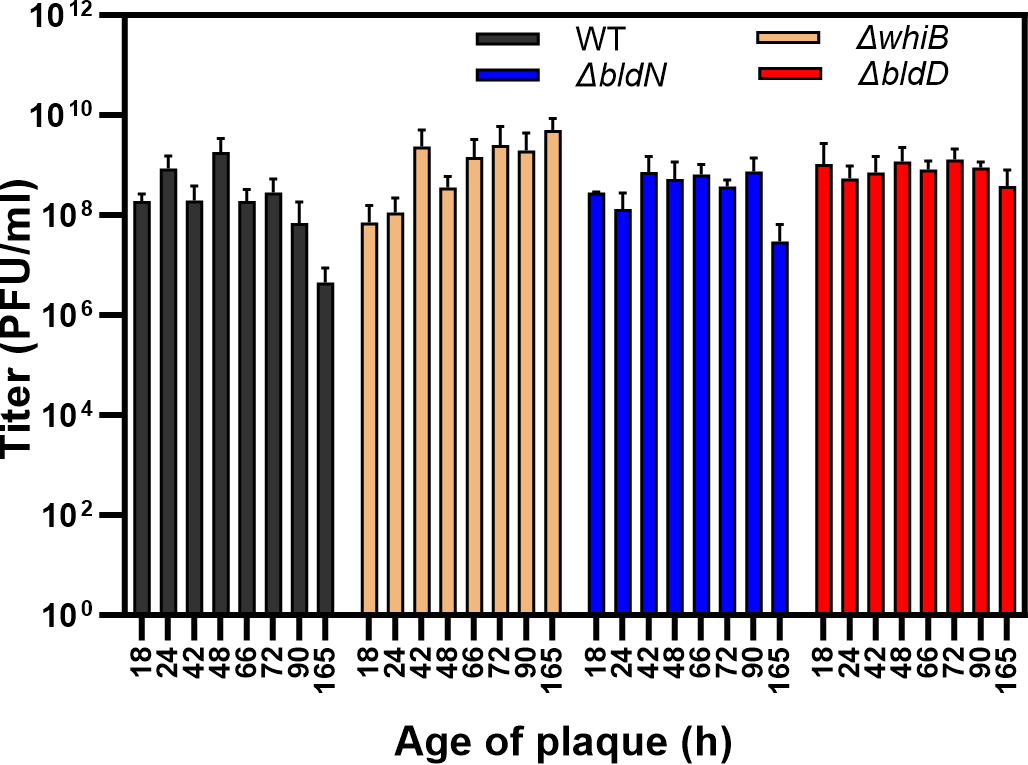


**Supplementary Figure S2: Re-infection of mycelium from plaque interfaces.** Samples were taken from plaque interfaces of differently aged plaques (n=3 for each strain and time point) and cultivated for 24 h in fresh medium before being used for double agar overlay assays.


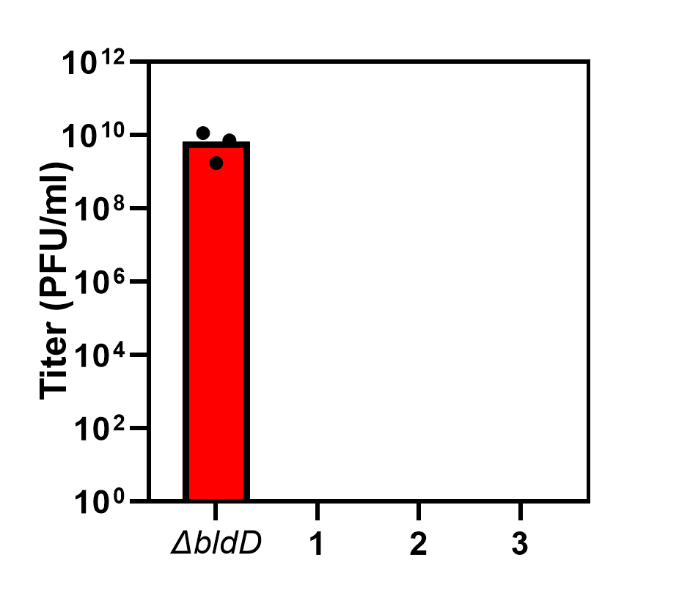


**Supplementary Figure S3: Plaque count of Alderaan on ΔbldD mycelium regrown from liquid infections.** Alderaan titer on S. venezuelae ∆bldD::apr (∆bldD) and on the three regrown cultures during liquid infection harvested after 70 h (1, 2, 3). Spotting was performed in triplicates.


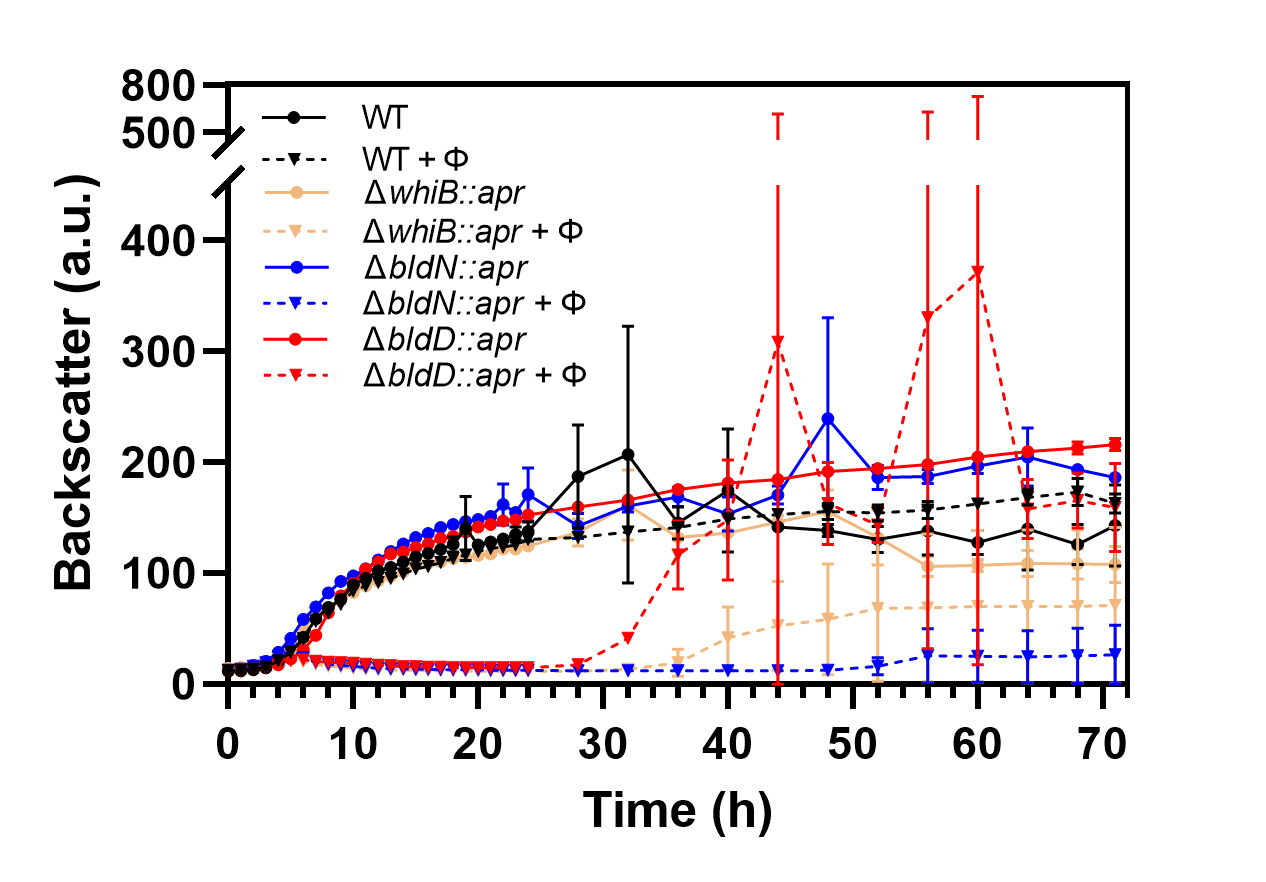


**Supplementary Figure S4: Liquid infection of S. venezuelae wild type, ΔwhiB, ΔbldN and ΔbldD with Alderaan.** Infection was performed and growth was measured in the BioLector microcultivation system with an initial titer of 10^7^ PFU/ml (n=3).


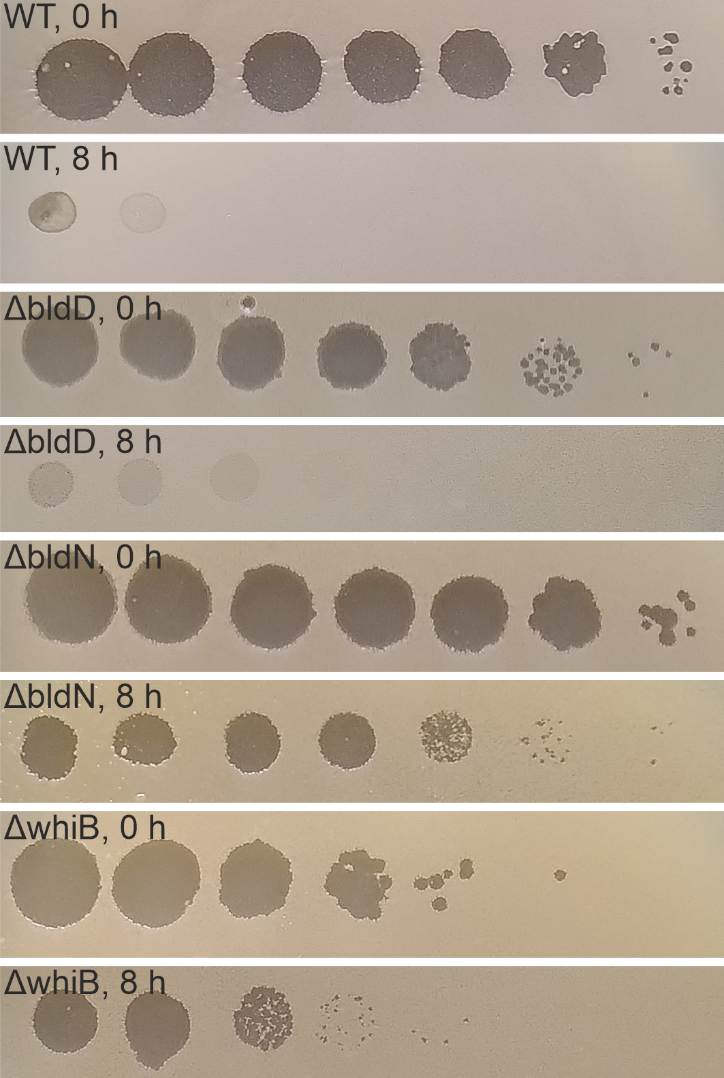


**Supplementary Figure S5: Plaques of Alderaan on aged mycelium of S. venezuelae.** Shown are representative plaques; further details are provided in Figure 6A.


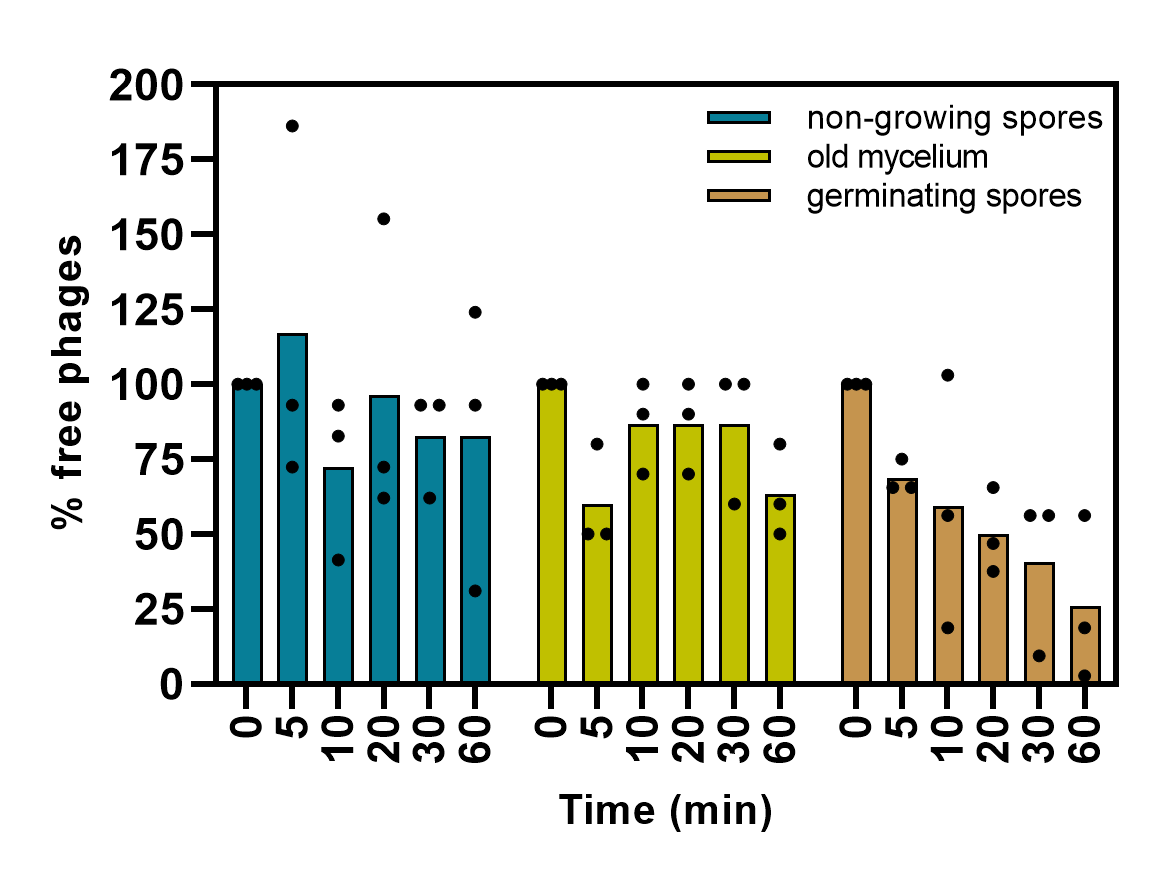


**Supplementary Figure S6: Adsorption of phage Alderaan to S. venezuelae.** 10^7^ PFU/ml of phage Alderaan were added to different developmental stages of S. venezuelae liquid cultures. Spores were inoculated in SM buffer (turquois, non-growing) and GYM medium (brown, germinating) at a final concentration of 10^8^ spores/ml (= OD_450_ of 0.15) and incubated for 4 h at 30 °C and 170 rpm. A mycelial stock from an overnight culture was used to inoculate GYM medium at a final OD_450_ of 0.15 and was grown for 24 h (yellow, old mycelium). Samples were taken at the indicated time points, centrifuged and supernatants were spotted to determine phage titers of biological triplicates and plot the percentage of free phages.


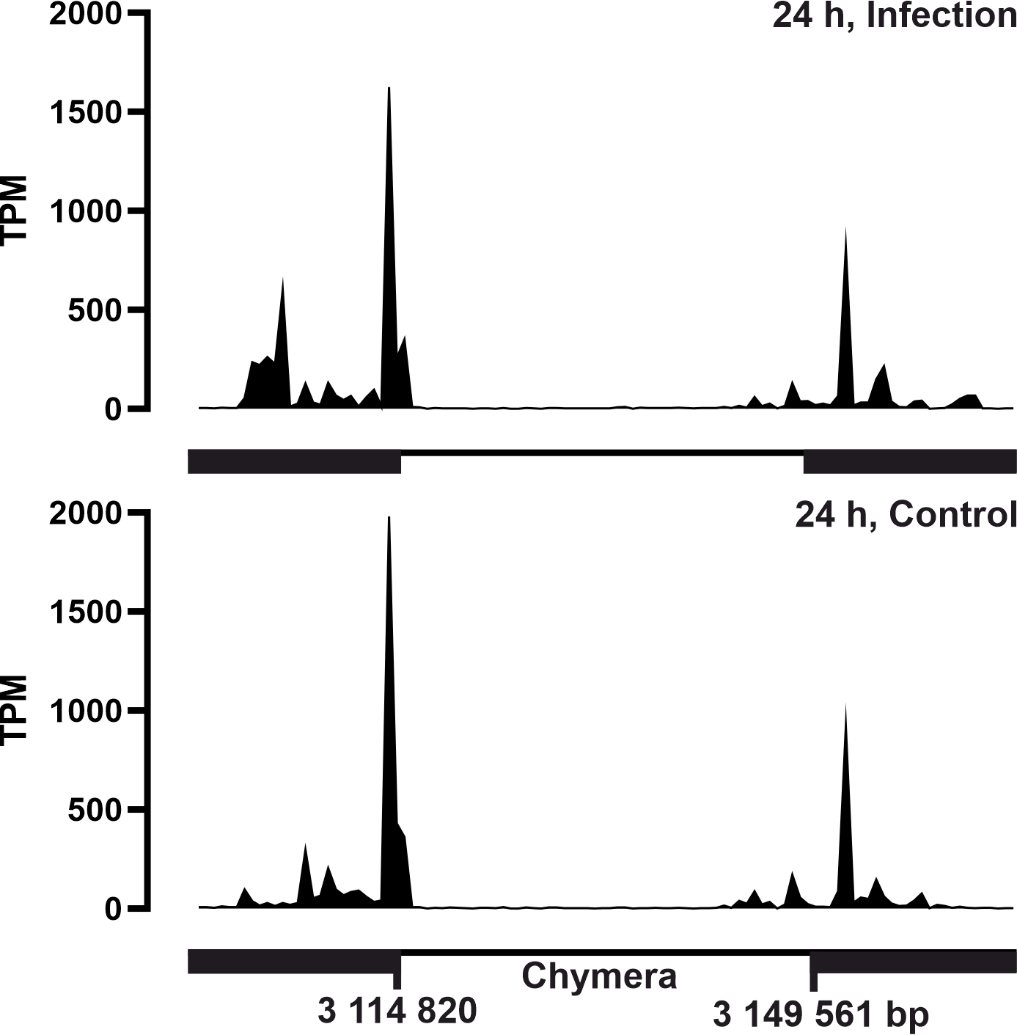


**Supplementary Figure S7: Expression of the Chymera genome during infection of S. venezuelae.** RNA-seq analysis of biological duplicates. Expression in transcripts per million (TPM) taken at 24 h from infected and uninfected (control) samples.


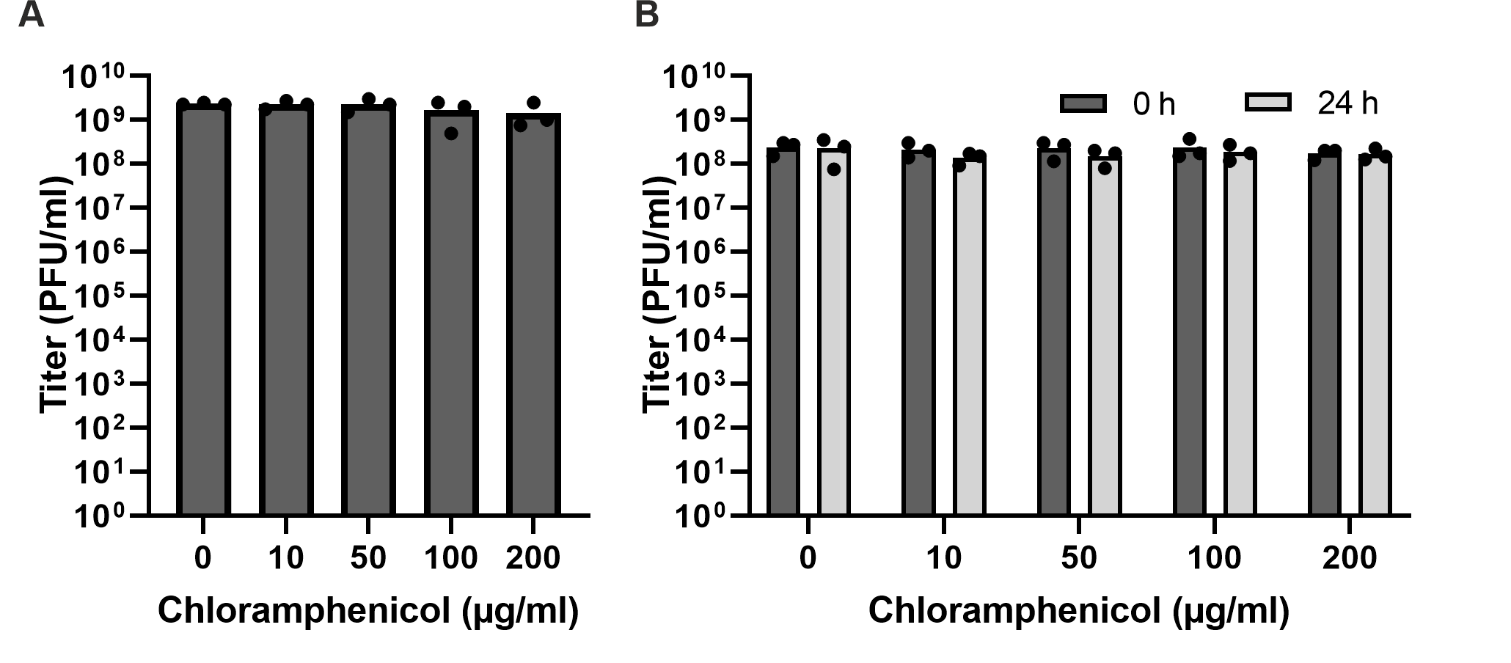


**Supplementary Figure S8: Plaque formation of Alderaan in presence of chloramphenicol.** The titer in PFU/ml was measured with Alderaan infecting S. venezuelae WT. **(A)** Different chloramphenicol concentrations were added to the bottom as well as the top agar. **(B)** Chloramphenicol was added to phage-containing SM buffer and incubated for 0 or 24 h at 30 °C and 900 rpm before spotting.

**References**

Bibb MJ, Domonkos A, Chandra G *et al.* Expression of the chaplin and rodlin hydrophobic sheath proteins in Streptomyces venezuelae is controlled by σ(BldN) and a cognate anti-sigma factor, RsbN. *Mol Microbiol* 2012;**84**:1033–49.

Bush MJ, Chandra G, Bibb MJ *et al.* Genome-Wide Chromatin Immunoprecipitation Sequencing Analysis Shows that WhiB Is a Transcription Factor That Cocontrols Its Regulon with WhiA To Initiate Developmental Cell Division in Streptomyces. *MBio* 2016;**7**:e00523-16.

Gomez-Escribano JP, Holmes NA, Schlimpert S *et al.* Streptomyces venezuelae NRRL B-65442: genome sequence of a model strain used to study morphological differentiation in filamentous actinobacteria. *J Ind Microbiol Biotechnol* 2021, DOI: 10.1093/jimb/kuab035.

Gregory MA, Till R, Smith MCM. Integration site for Streptomyces phage phiBT1 and development of site-specific integrating vectors. *J Bacteriol* 2003;**185**:5320–3.

Hardy A, Sharma V, Kever L *et al.* Genome Sequence and Characterization of Five Bacteriophages Infecting Streptomyces coelicolor and Streptomyces venezuelae: Alderaan, Coruscant, Dagobah, Endor1 and Endor2. *Viruses* 2020;**12**:1065.

Hong H-J, Hutchings MI, Hill LM *et al.* The Role of the Novel Fem Protein VanK in Vancomycin Resistance in Streptomyces coelicolor. *J Biol Chem* 2005;**280**:13055–61.

Kieser T, Bibb M, Chater K *et al.* *Practical Streptomyces Genetics: A Laboratory Manual*. Norwich: John Innes Foundation, 2000.

Liu Z, Shi Y, Zhang Y *et al.* Classification of Streptomyces griseus (Krainsky 1914) Waksman and Henrici 1948 and related species and the transfer of “Microstreptospora cinerea” to the genus Streptomyces as Streptomyces yanii sp. nov. *Int J Syst Evol Microbiol* 2005;**55**:1605–10.

MacNeil DJ, Gewain KM, Ruby CL *et al.* Analysis of Streptomyces avermitilis genes required for avermectin biosynthesis utilizing a novel integration vector. *Gene* 1992;**111**:61–8.

Stuttard C. Transduction of auxotrophic markers in a chloramphenicol-producing strain of Streptomyces. *J Gen Microbiol* 1979;**110**:479–82.

Tschowri N, Schumacher MA, Schlimpert S *et al.* Tetrameric c-di-GMP mediates effective transcription factor dimerization to control Streptomyces development. *Cell* 2014;**158**:1136–47.
